# *PADI4* Polymorphisms Confer Risk of Anti-CCP-Positive Rheumatoid Arthritis in Synergy With HLA-DRB1*04 and Smoking

**DOI:** 10.3389/fimmu.2021.707690

**Published:** 2021-10-18

**Authors:** Laura Massarenti, Christian Enevold, Dres Damgaard, Niels Ødum, Peter Garred, Morten Frisch, Miriam A. Shelef, Søren Jacobsen, Claus Henrik Nielsen

**Affiliations:** ^1^ Institute for Inflammation Research, Center for Rheumatology and Spine Diseases, Section 7521, Copenhagen University Hospital Rigshospitalet, Copenhagen, Denmark; ^2^ LEO Foundation Skin Immunology Research Center, Department of Immunology and Microbiology, University of Copenhagen, Copenhagen, Denmark; ^3^ Laboratory of Molecular Medicine, Department of Clinical Immunology, Section 7631, Copenhagen University Hospital Rigshospitalet, Copenhagen, Denmark; ^4^ Department of Epidemiology Research, Statens Serum Institut, Copenhagen, Denmark; ^5^ Department of Medicine, University of Wisconsin–Madison, Madison, WI, United States; ^6^ William S. Middleton Memorial Veterans Hospital, Madison, WI, United States; ^7^ Copenhagen Lupus and Vasculitis Clinic, Center for Rheumatology and Spine Diseases, Section 4242, Copenhagen University Hospital Rigshospitalet, Copenhagen, Denmark; ^8^ Department of Odontology, Faculty of Health and Medical Sciences, University of Copenhagen, Copenhagen, Denmark

**Keywords:** rheumatoid arthritis, peptidylarginine deiminase (PAD), anti-citrullinated proteins antibodies (ACPA), HLA-DRB1*04, single nucleotide polymorphism (SNP)

## Abstract

Peptidylarginine deiminases (PADs) catalyze citrullination, a post-translational modification playing a pathogenic role in anti-citrullinated protein antibody (ACPA)-positive rheumatoid arthritis (RA). The interplay between single nucleotide polymorphisms (SNPs) in the *PADI* genes and known risk factors for ACPA-positive RA, including smoking, HLA-DR4 and -1, and the PTPN22 R620W polymorphism, was investigated. We typed four *PADI2* SNPs, four *PADI4* SNPs, and the PTPN22 R620W SNP in 445 Danish RA patients and 533 age-matched healthy controls, as well as in 200 North American RA patients and 100 age- and sex-matched controls. The *HLA-DRB1* locus was typed in the Danish cohort. Logistic regression analyses, adjusted for age, sex, smoking status, and PTPN22 R620W, revealed increased risk of anti-CCP-positive RA in carriers of rs11203367(T) (OR: 1.22, p=0.03) and reduced risk in carriers of rs2240335(A) in *PADI4* (OR: 0.82, p=0.04). rs74058715(T) in *PADI4* conferred reduced risk of anti-CCP-negative RA (OR: 0.38, p=0.003). In HLA-DRB1*04-positive individuals, specifically, the risk of anti-CCP-positive RA was increased by carriage of *PADI4* rs1748033(T) (OR: 1.54, p=0.007) and decreased by carriage of *PADI4* rs74058715(T) (OR: 0.44, p=0.01), and we observed an interaction between these SNPs and HLA-DRB1*04 (p=0.004 and p=0.008, respectively) Thus, *PADI4* polymorphisms associate with ACPA-positive RA, particularly in HLA-DRB1*04-positive individuals, and with ACPA-negative RA independently of *HLA*-DRB1*04.

## Introduction

Peptidylarginine deiminases (PADs) catalyze the post-translational conversion of peptidylarginine to peptidylcitrulline. Citrullination is involved in many physiological functions, including skin keratinization, neuron myelination, and formation of neutrophil extracellular traps (NETs) (1–3) but is also thought play a pathogenic role in anti-citrullinated protein-antibody (ACPA)-positive rheumatoid arthritis (RA) (4). ACPAs, routinely detected using the anti-cyclic citrullinated peptide (anti-CCP) test (5), are often present years before clinically overt disease (6), and anti-CCP-positive RA patients have a worse prognosis with a higher degree of erosive damage than anti-CPP-negative RA patients (7). ACPA-positive RA is strongly associated with HLA molecules containing the so-called shared epitope (SE), which are capable of binding certain citrullinated peptides (8–10). The shared epitope is present in most HLA-DRB1*04 subtypes (HLA-DRB1*0401, HLA-DRB1*0404, HLA-DRB1*0405, and HLA-DRB1*0408 but not in HLA-DR4B1*0402) and HLA-DRB1*01 subtypes (HLA-DRB1*0101 and HLA-DRB1*0102). However, HLA-DRB1*04, which is carried by 67–78% of anti-CCP-positive RA patients (10–12), correlates more strongly with anti-CCP-positive RA than HLA-DRB1*01 (reported in 22–51% of anti-CCP-positive RA patients) (10–12). In addition, the R620W variant of protein tyrosine phosphatase, non-receptor type 22 (PTPN22), confers increased risk of ACPA-positive RA (13, 14), as does smoking (9).

The five existing PAD isoforms in humans are encoded by the *PADI1-4* and *-6* genes (15). PAD2 and PAD4 are expressed by immune cells (16–18) and are present in the synovium of RA patients, where their expression levels correlate with those of inflammatory markers (18, 19). Some investigators have found that PAD4 generates autoantigens recognized by ACPAs more efficiently than PAD2 (20), while others have shown that the two PAD isoforms are approximately equally efficient (21).

**Figure 1:**
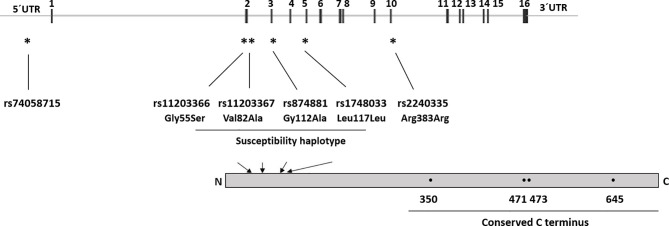
*PADI4* gene structure and SNP location. UTR, Untranslated region, 1–16 indicates the exon number. * indicates SNPs location within the *PADI4* gene, arrows indicate location of the SNP corresponding amino acids in the protein, and • indicates active site residues (Asp350, His471, Asp473, and Cys645).

Studies on Asian populations have shown associations between RA and single nucleotide polymorphisms (SNPs) in *PADI2* (22, 23) as well as SNPs in *PADI4* (22–27). However, the observed associations with *PADI2* SNPs in Asian populations have not been replicated in studies on European or North American cohorts, while a few studies have confirmed the association of the *PADI4* SNPs with RA in those populations (28–34). Notably, one study reported associations between one SNP in PADI4 (rs2240340) and RA in a North American cohort, alone or when combined with a Swedish cohort, but not in the Swedish cohort alone (31). A haplotype of *PADI4* has been reported to confer susceptibility to RA in Asians (24), but not in a British study (30). The haplotype consists of the minor alleles of rs11203366, rs11203367, and rs874881 encoding the amino acid substitutions Gly55Ser, Val82Ala, and Gly112Ala, respectively, and rs1748033 which is a synonymous SNP (**Figure 1**). Besides, the minor allele (A) of the synonymous SNP rs2240035 has been shown to decrease the risk of RA in Asians and North Americans (26, 27, 35). Together with rs74058715, which is located in the 5’ untranslated region (UTR) of *PADI4*, rs2240035 has been shown to regulate PAD4 expression levels in neutrophils and monocytes (36).

Only few studies on *PADI* polymorphism in RA have stratified the patients according to HLA types (28, 32, 37, 38) and, generally, studies addressing the influence of HLA types on development of RA have not taken *PADI* polymorphism into account. We hypothesized that *PADI* polymorphisms exert their greatest influence in subjects carrying HLA types that bind citrullinated peptides most strongly, i.e., HLA-DRB1*04.

Our aim was to determine if anti-CCP-positive or anti-CCP-negative RA is associated with SNPs in *PADI2* or *PADI4* in a Danish and a North American cohort of RA patients and healthy controls. Moreover, we aimed at elucidating the interaction of *PADI* SNPs with HLA-DRB1*04 or HLA-DRB1*01 in predisposing to RA.

## Materials and Methods

### Patients and Controls

The Danish cohort included 445 RA patients clinically identified at 21 rheumatology departments across Denmark and 533 population controls matched for age (**Table 1**). The cohort has previously been reported on (39). The RA patients had less than 5 years of disease duration and fulfilled the American College of Rheumatology 1987 classification criteria for RA (40). Healthy controls, frequency-matched by birth year, were randomly selected from the Danish population, and blood samples were collected by general practitioners. Written informed consent was obtained from all study subjects, and the study was approved by the Scientific Ethical Committees for Copenhagen and Frederiksberg (KF 01-039/01), the Danish Data Protection Agency (2001–41–0658), and the Institutional Review Board at Statens Serum Institut (21–00050). The North American cohort included 200 RA patients and 100 age- and sex-matched controls without autoimmune disease selected from the University of Wisconsin (UW) Rheumatology Biorepository described in (35, 41) (**Table 1**). The RA patients were initially identified by having at least two outpatient visits with RA-associated ICD codes within 24 months (42), or one visit and a positive anti-CCP test. Diagnosis was confirmed by manual review of rheumatology notes. All subjects gave written informed consent, and the study was approved by the Institutional Review Board of the University of Wisconsin-Madison (#2015-0156).

**Table 1:** Demographic, clinical, and genetic characteristics of anti-CCP-positive and anti-CCP-negative rheumatoid arthritis (RA) patients and controls.

	Danish cohort (n= 978)			North American cohort (n= 300)		
	Anti-CCP- positive RA(n = 309)	Anti-CCP- negative RA(n = 136)	Controls(n = 533)	Anti-CCP- positive RA(n = 104)	Anti-CCP- negative RA(n = 96)	Controls(n =100)
Age, median (range)	52 (19–68)	53 (21–69)	53 (19–69)	64 (38–87)	63 (34–90)	63 (33–95)
Women, n (%)	209 (68%)	104 (76%)	327 (61%)	72 (69%)	83 (86%)	79 (79%)
Ever smokers, n (%)	226 (73%)	85 (62%)	323 (61%)	57 (55%)	46 (48%)	39 (39%)
Any PTPN22 R620W, n (%)	98 (32%)	33 (24%)	108 (20%)	26 (25%)	28 (29%)	22 (22%)
Rheumatoid factor IgM, n (%)	275 (89%)	40 (29%)	64 (12%)	76 (73%)^‡^	30 (31%)^‡^	NA
Any HLA-DRB1*04, n (%)	227 (73%)	50 (37%)	193 (36%)	NA	NA	NA
Any HLA-DRB1*01, n (%)	78 (25%)	28 (21%)	102 (19%)	NA	NA	NA
No HLA-DRB1*04 or HLA-DRB1*01, n (%)	47 (15%)	64 (47%)	257 (48%)	NA	NA	NA
Caucasian ethnicity, n (%)	(99%)	(99%)	(99%)	(93%)	(96%)	(94%)

CCP, cyclic citrullinated peptide; RA, rheumatoid arthritis; HLA, human leukocyte antigen; NA, not available.

^‡^info missing for one anti-CCP-positive and seven anti-CCP-negative subjects.

### SNP Selection

SNPs in *PADI2* (rs2057094, rs2076616, rs2235912, and rs1005753) and *PADI4* (rs74058715, rs11203367, rs1748033, and rs2240335) were selected based on literature search of previously reported associations with RA (22–27, 43). rs11203367 was chosen as a tag-SNP for the two other SNPs encoding amino acid substitutions in the RA susceptibility haplotype (rs11203366 and rs874881), given the strong linkage disequilibrium (LD) between these three SNPs (44). Additionally, as previously shown, rs11203367 is also in strong LD (r^2^ > 0.8), with the intronic SNPs rs2240340 and rs11203368 (44), two other SNPs associated with RA in previous studies (23, 31); therefore these intron located SNPs were excluded from typing. The *PTPN22* missense SNP rs2476601 (R620W) was also included, given the reported association with RA and its known interactions with other risk factors like smoking and carriage of the SE (14). None of the SNPs investigated deviated significantly from Hardy-Weinberg equilibrium, in either cases or controls.

### Genotyping

DNA was extracted from whole blood samples from the two cohorts as previously described (35, 39) and stored at −80°C until use. Samples were genotyped for *PADI* SNPs and the rs2476601 SNP in *PTPN22*, encoding R620W, by means of an in-house multiplex SNP assay protocol, as previously described (45). In brief, the method included a polymerase chain reaction (PCR) to amplify the selected SNP sites and an allele-specific primer extension (ASPE) reaction for labeling ASPE-oligonucleotides, followed by hybridization to MagPlex-TAG™ bead sets (Luminex Corporation, Austin, TX, USA) for analysis on the Luminex platform (Luminex Corporation, Austin, TX, USA). All assay runs included a panel of control samples with known genotypes (Coriell Cell Repository, Camden, NJ, USA) as well as no template negative controls. Low-resolution *HLA-DRB1* typing of the Danish cohort had been performed prior to this study, according to the 11^th^ Histocompatibility Workshop protocol (46).

### Statistical Analysis

Hardy-Weinberg equilibrium was calculated with the “hwde”-package in R (R Foundation for Statistical Computing, Vienna, Austria). Associations between SNPs and disease were tested using multiple logistic regressions with adjustment for age, sex, smoking status, and carriage of PTPN22 R620W. P-values and odds ratios (ORs) with 95% confidence intervals (95% CI) were calculated for minor allele counts (trend test). Analyses of the Danish and North American cohorts were performed separately and, in order to increase statistical power, repeated after merging of the two cohorts. *Post-hoc* analyses after stratification for *HLA-DRB1* alleles were performed in the Danish cohort, with further adjustment for the number of HLA-DRB1*04 or HLA-DRB1*01 alleles carried. Disease risk due to combined effects of PTPN22 R620W, *HLA-DRB1* alleles, *PADI4* alleles, and smoking was calculated by means of logistic-regression models, with adjustment for age and sex. Gene-gene interaction and gene-exposure interaction were calculated by logistic regression analyses including all single variables and interaction variables in the regression model. A full factorial set of interactions was presented to the model and only conditionally included if they significantly improved the predictive performance of the model. Analyses were performed in R Studio Version 1.0.153 (RStudio Inc., Boston, MA, USA) using R version 3.4.2 (R Foundation for Statistical Computing, Vienna, Austria) and in SPSS 25.0 (IBM, New York, USA). The level of statistical significance was set at p<0.05.

## Results

The characteristics of the Danish and North American cohorts included in this study are shown in **Table 1**. The North American cohort had a median age of 63 years (range 33–95), which was higher than the 53 years (range 19–69) of the Danish cohort, and it contained more women than the Danish cohort, particularly within the anti-CCP-negative RA and healthy control groups. Anti-CCP-positive RA patients were more often smokers than anti-CCP-negative patients in both cohorts, but the Danish cohort contained more ever smokers than the North American cohort in all three study groups. All analyses were adjusted for age, sex, smoking status, and cohort origin.

### Associations Between PADI Polymorphisms and RA

We studied associations of the *PADI* SNPs with anti-CCP-positive and anti-CCP-negative RA separately. Given that carriage of PTPN22 R620W is a known risk factor for anti-CCP-positive RA (14), we adjusted all analyses for this variable too. To increase statistical power, we merged the two cohorts in these analyses but adjusted the analyses for the origin of the cohort. We found that none of the *PADI2* SNPs included in this study associated with anti-CCP-positive or anti-CCP-negative RA (**Table 2**).

**Table 2:** Associations between selected polymorphisms in the *PADI* genes and anti-CCP-positive or anti-CCP-negative RA.

	Minor allele	Controls (n = 633)			Anti-CCP-positive RA (n = 413)							Anti-CCP-negative RA (n = 232)						
		pp	pq	qq	pp	pq	qq		OR	95% CI	p-value	pp	pq	qq		OR	95% CI	p-value
***PADI2***																		
rs2057094	C	242	289	102	170	175	68		1.00	[0.83;1.19]	0.97	78	111	43		1.14	[0.92;1.44]	0.23
rs2076616	C	312	260	61	213	161	39		0.98	[0.81;1.19]	0.85	101	102	29		1.19	[0.94;1.50]	0.15
rs2235912	C	231	301	101	170	190	53		0.88	[0.73;1.06]	0.17	83	110	39		1.06	[0.84;1.33]	0.62
rs1005753	G	240	303	90	137	220	56		1.12	[0.92;1.35]	0.24	93	107	32		0.97	[0.77;1.22]	0.79
***PADI4***																		
rs74058715	T	553	79	1	369	43	1		0.85	[0.57;1.26]	0.41	220	12	0		**0.38**	**[0.20;0.73]**	**0.003**
rs11203367	T	218	309	106	121	205	87		**1.22**	**[1.02;1.47]**	**0.03**	64	136	32		1.09	[0.86;1.38]	0.47
rs1748033	T	297	281	55	178	186	49		1.18	[0.97;1.44]	0.09	92	119	21		1.16	[0.91;1.48]	0.24
rs2240335	A	267	297	69	199	175	39		**0.82**	**[0.67;0.99]**	**0.04**	108	111	13		**0.78**	**[0.61;1.00]**	**0.05**

CCP, Cyclic Citrullinated Peptide; RA, Rheumatoid Arthritis; p, major allele; q, minor allele; OR, Odds Ratio; 95%CI, 95% confidence interval. Values marked in bold indicate 95% CI excluding 1.00 and p<0.05. Logistic regression with adjustment for age, sex, ever smoking and carriage of PTPN22 R620W.

Among the *PADI4* SNPs examined, the minor allele (T) of rs11203367 [the tag-SNP for the previously reported RA susceptibility haplotype (24, 30)], was associated with an increased risk of anti-CCP-positive RA (OR: 1.22, p=0.03) (**Table 2**). The minor allele (A) of rs2240335, on the other hand, appeared to protect against both anti-CCP-positive RA (OR: 0.82, p=0.04) and anti-CCP-negative RA (OR: 0.78, p=0.05). Moreover, the minor allele (T) of rs74058715 appeared to protect against anti-CCP-negative RA (OR: 0.38, p=0.003) (**Table 2**).

The majority of these reported associations was largely driven by the North American cohort, with exception of the association of rs74058715 and anti-CCP-negative RA (**Supplementary Table 1**).

### Association of PADI Polymorphisms With RA by HLA-Type

The Danish cohort was HLA-typed, so we reanalyzed their data, taking this variable into account. We chose to examine the influence of HLA-DRB1*04 and HLA-DRB1*01 separately, since HLA-DRB1*04 is more strongly associated with anti-CCP-positive RA than HLA-DRB1*01 (12) (subjects were grouped as illustrated in **Supplementary Figure 1**; of note, subjects carrying both alleles were included in the analysis of HLA-DRB1*04).

**Figure 2:**
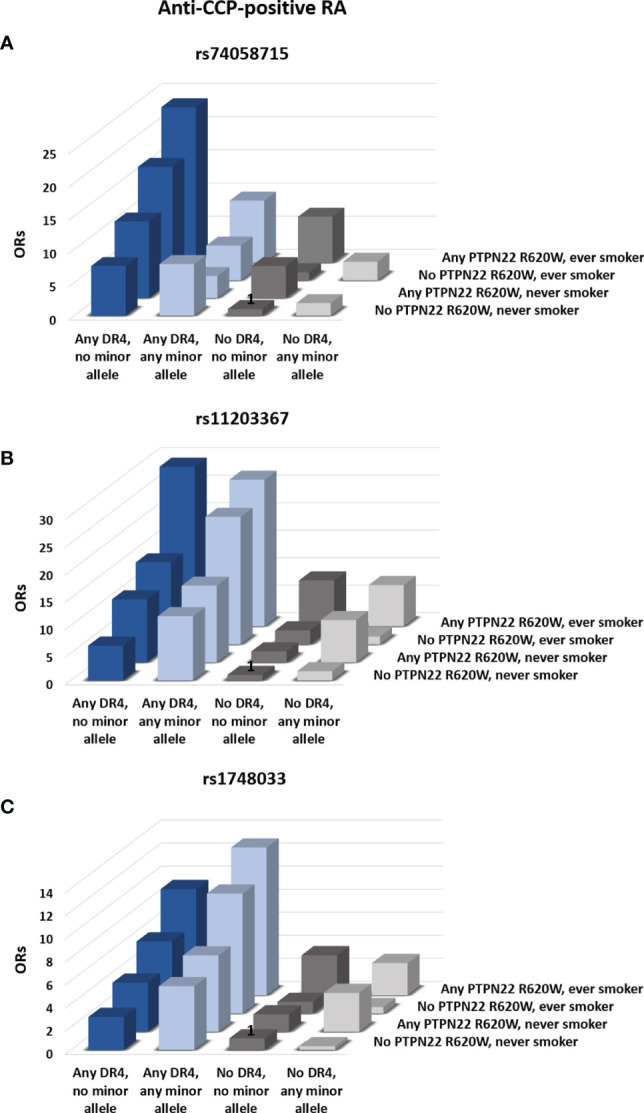
Influence of *PADI4* SNPs on the risk of RA. The influence of the minor alleles of rs74058715 **(A)**, rs11203367 **(B)**, and rs1748033 **(C)** in *PADI4* on the cumulative risk of anti-CCP–positive RA was assessed. Bars represent odds-ratios (ORs) for different combinations of absence or presence of any copy of the minor allele of the three *PADI4* SNPs, any HLA-DRB1*04 allele, any PTPN22 R620W, and smoking. Subjects who carried HLA-DRB1*01 in combination with other HLA types than HLA-DRB1*04 were excluded from the analysis. Bars marked with “1” served as the reference condition. All ORs are adjusted for age and sex. ORs and p-values for all comparisons are reported in **Supplementary Table 3**.

Inclusion of HLA-DR type in the analysis did not influence the overall conclusions with respect to SNPs in *PADI2* (data not shown). In HLA-DRB1*04-positive subjects, on the other hand, the minor allele (T) of rs74058715 was associated with a significantly reduced risk of anti-CCP-positive RA after adjustment for age, sex, smoking status, PTPN22 R620W, and number of HLA-DRB1*04 alleles (p=0.01; **Table 3**). This effect can be observed in **Figure 2A**, which shows the cumulative ORs associated with different combinations of four risk factors (HLA-DRB1*04 alleles, *PADI4* alleles, PTPN22 R620W and smoking) compared to the reference group without any of these risk factors. Hence, the ORs for HLA-DRB1*04-positive subjects who carried the minor allele (light blue bars) were generally lower than the ORs for HLA-DRB1*04-positive subjects who did not carry this allele (dark blue bars). By contrast, the minor allele (T) of rs11203367 conferred a borderline significant increase in OR for anti-CCP-positive RA in HLA-DRB1*04-positive subjects (p=0.06, **Table 3**), as visualized in **Figure 2B** (light blue bars for carriers *versus* dark blue bars for non-carriers). Notably, rs1748033(T), which belongs to the same RA susceptibility haplotype as rs11203367(T), associated strongly with anti-CCP-positive RA in HLA-DRB1*04-positive individuals (p=0.007; **Table 3**), with higher cumulative ORs for carriers of this allele compared to non-carriers (**Figure 2C**, light blue bars *vs* dark blue bars).

**Table 3:** Association of *PADI4* SNPs with RA by HLA-type.

	Controls (n = 533)			Anti-CCP-positive RA (n = 309)						Anti-CCP-negative RA (n = 136)					
	pp	pq	qq	pp	pq	qq	OR	95% CI	p-value	pp	pq	qq	OR	95% CI	p-value
**rs74058715**															
HLA-DRB1*04^+^ ^1^	161	32	0	207	20	0	**0.44**	**[0.23;0.84]**	**0.01**	47	3	0	0.31	[0.09;1.08]	0.06
HLA-DRB1*01^+^ ^2^	75	8	0	29	6	0	2.35	[0.69;8.01]	0.17	21	1	0	0.58	[0.06;5.13]	0.62
Other HLA-types	229	27	1	42	5	0	1.02	[0.37;2.85]	0.96	60	4	0	0.51	[0.17;1.49]	0.22
**rs11203367**															
HLA-DRB1*04^+^ ^1^	71	92	30	65	115	47	1.32	[0.99;1.77]	0.06	12	33	5	1.11	[0.67;1.82]	0.69
HLA-DRB1*01^+^ ^2^	26	40	17	11	20	4	0.70	[0.38;1.30]	0.26	7	14	1	0.72	[0.34;1.52]	0.39
Other HLA-types	87	121	49	17	21	9	1.07	[0.68;1.70]	0.76	18	36	10	1.05	[0.71;1.55]	0.82
**rs1748033**															
HLA-DRB1*04^+^ ^1^	105	74	14	94	104	29	**1.54**	**[1.13;2.12]**	**0.007**	20	28	2	1.28	[0.75;2.16]	0.36
HLA-DRB1*01^+^ ^2^	35	39	9	15	17	3	0.78	[0.40;1.50]	0.46	12	9	1	0.64	[0.29;1.44]	0.29
Other HLA-types	110	124	23	27	15	5	0.85	[0.50;1.43]	0.54	23	35	6	1.20	[0.78;1.84]	0.41
**rs2240335**															
HLA-DRB1*04^+^ ^1^	77	96	20	106	98	23	0.81	[0.59;1.11]	0.18	21	26	3	0.91	[0.53;1.54]	0.72
HLA-DRB1*01^+^ ^ 2^	37	36	10	20	11	4	0.78	[0.41;1.49]	0.46	6	16	0	1.16	[0.55;2.47]	0.70
Other HLA-types	116	117	24	20	20	7	1.13	[0.69;1.83]	0.63	31	28	5	0.90	[0.58;1.38]	0.63

CCP, cyclic citrullinated peptide; RA, rheumatoid arthritis; HLA, human leukocyte antigen; p, major allele; q, minor allele; OR, odds ratio; 95% CI, 95% confidence interval. Logistic regression with adjustment for age, sex, ever smoking, carriage of PTPN22 R620W, and carriage of HLA-DRB1*04 or HLA-DRB1*01. Values marked in bold indicate 95% CI excluding 1.00 and p<0.05.

^1^Including heterozygotes carrying both HLA-DRB1*04 and HLA-DRB1*01; adjusted for the number of HLA-DRB1*04 alleles.

^2^Excluding heterozygotes carrying both HLA-DRB1*04 and HLA-DRB1*04; adjusted for the number of HLA-DRB1*01 alleles.

No clear pattern was observed for HLA-DRB1*01-positive individuals or for subjects who carried neither HLA-DRB1*04 nor HLA-DRB1*01 (**Table 3** and gray bars in **Figure 2**). Neither were any associations between SNPs in *PADI* and anti-CCP-negative RA revealed after adjustment for HLA type (**Table 3**).

### Gene-Gene and Gene-Exposure Interactions

In view of the associations between SNPs in *PADI4* and anti-CCP-positive RA and the known interaction between the SE and smoking as risk factors for anti-CCP-positive RA, we analyzed the interactions between *PADI4* SNPs and other risk factors, i.e., HLA-DRB1*04, HLA-DRB1*01, PTPN22 R620W, and smoking on the risk of anti-CCP-positive or anti-CCP-negative RA.

We observed a strong interaction between the major allele (C) of rs74058715, HLA-DRB1*04 and ever-smoking, that associated with increased risk of anti-CCP-positive RA (p=0.008, **Supplementary Table 2**). Similarly, the minor allele (T) of rs1748033 and HLA-DRB1*04 interacted synergistically increasing the risk of anti-CCP-positive RA, irrespective of smoking status (p=0.004, **Supplementary Table 2**). Regarding the risk of anti-CCP-negative RA, we also observed an interaction between the minor allele (T) of rs1748033, HLA-DRB1*04 and ever-smoking (p=0.05, **Supplementary Table 2**).

## Discussion

PAD2 and PAD4 are thought to be involved in the pathogenesis of ACPA-positive RA by catalyzing the formation of citrullinated autoantigens that are recognized by T cells and targeted by ACPAs (8). However, citrullination by PADs may have other potentially pathogenic roles, including regulation of T-cell responses  (47, 48) and induction of NET formation (3, 49). Accordingly, SNPs in *PADI2* and *PADI4* have been shown to associate with RA, mainly in Asian populations (22–27).

This study did not show significant associations between *PADI2* SNPs and RA in the two mainly Caucasian cohorts examined. We cannot rule out, however, that the minor allele (C) of rs2235912 confers resistance to anti-CCP-positive RA, as suggested by a weak tendency in that direction, which is in agreement with findings from a previous meta-analysis (43).

After merging the Danish and North American cohorts and adjusting the model for the origin of the cohorts, we found that the minor allele (T) of rs11203367 in *PADI4* (tag-SNP for a haplotype containing Gly55Ser, Val82Ala, and Gly112Ala) associated with anti-CCP-positive RA with an OR of 1.22. The association was largely driven by the North American cohort, in accordance with a previous study showing association with rs2240340, which is in high LD with the SNP in our study (31). Furthermore, the minor allele (A) of the exon-synonymous SNP rs2240335 was associated with a decreased risk of anti-CCP-positive RA with an OR of 0.82, in agreement with previous results (35).

An especially strong association was observed in HLA-DRB1*04-positive subjects in the Danish cohort between the minor allele (T) of rs1748033 and anti-CCP-positive RA, and a similar tendency was observed for rs11203367, which is also part of RA the susceptibility haplotype. In individuals carrying HLA-DRB1*04, the minor (T) allele of rs74058715 was also associated with reduced risk of anti-CCP-positive RA with an OR of 0.44. This SNP is not part of the RA susceptibility haplotype but is associated with decreased expression of PAD4 in neutrophils and monocytes (36). These results suggest that SNPs in *PADI4* alter the cumulative risk of disease conferred by other well-characterized risk factors (HLA-DRB1*04, PTPN22 R620W, and smoking). In accordance, we observed an interaction between the major allele (C) of rs74058715, HLA-DRB1*04, and ever-smoking, as well as between the minor allele (T) of rs1748033 and HLA-DRB1*04 in increasing the risk of anti-CCP-positive RA.

Somewhat unexpectedly, we observed that the minor (T) allele of rs74058715 was associated with reduced risk of anti-CCP-negative RA, and the minor allele (A) of rs2240335 showed a similar tendency. Furthermore, we observed an interaction between the minor allele (T) of rs1748033, HLA-DRB1*04, and ever-smoking in conferring risk of anti-CCP-negative RA. Generation of citrullinated autoantigens does not explain a pathogenic role for citrullination in ACPA-negative RA, but *PADI* polymorphisms may be associated with differences in citrullination patterns that may have an adverse influence on the generation of pro-inflammatory mediators (50), regulation of T-cell responses (47, 48), or NET formation (3, 49) potentially leading to tissue damage.

Our study is limited by the relatively low number of subjects included and the lack of HLA-typing of the North American cohort. Furthermore, only low-resolution HLA-typing was performed so the HLA-DRB1*04-positive group may have included individuals carrying HLA-DRB1*0402, which does not contain the SE and has been associated with a protective effect against RA (51).

It is important to note that although HLA-DRB1*04 and HLA-DRB1*01 are often grouped together as SE-containing serotypes, we chose to separate them in our analysis, since the association of HLA-DRB1*04 with RA is considerably stronger than that of HLA-DRB1*01 (12). We found no association between SNPs in *PADI* and RA in HLA-DRB1*01-positive individuals, and no interaction between *PADI4* SNPs and HLA-DRB1*01, which may be due to the low power of the study. On the other hand, cohort stratifications and lack of correction due to the exploratory nature of the study may have led to type I errors. Thus, similar and more robust analyses in larger cohorts are warranted.

Our study suggests that certain SNPs in *PADI4* are risk factors for ACPA-positive RA, especially in HLA-DRB1*04-positive individuals. Furthermore, it shows that *PADI4* SNPs may also play a role in ACPA-negative RA.

## Data Availability Statement

The datasets generated during and/or analyzed during the current study are available from the corresponding author on reasonable request.

## Ethics Statement

The studies involving human participants were reviewed and approved by the Scientific Ethical Committees for Copenhagen and Frederiksberg (KF 01-039/01), the Danish Data Protection Agency (2001–41–0658), the Institutional Review Board at Statens Serum Institut (21–00050), and the Institutional Review Board of the University of Wisconsin–Madison (#2015-0156). The patients/participants provided their written informed consent to participate in this study.

## Author Contributions

LM, CN, DD, MS, MF, PG, SJ, and CE designed the study. LM and PG carried out the experiments. LM, CE, and SJ carried out the statistical analyses. LM, CN, and CE drafted the manuscript. SJ, MF, and MS provided the sample material and the clinical data for the Danish and North American cohorts, respectively. DD, NØ, and MS revised the manuscript critically. All authors contributed to the article and approved the submitted version.

## Funding

This study was provided by the Independent Research Fund, Denmark (grant number DFF - 7016-00233) to CN as well as from the University of Wisconsin School of Medicine and Public Health from the Wisconsin Partnership Program (grant number 2951) and the Doris Duke Charitable Foundation (grant number 2015099) to MS. SJ was supported by grants from the Danish Rheumatism Association (grant number A3865) and the Novo Nordisk Foundation. MS was additionally supported by NIH/NIAMS (grant number K08AR065500) and U.S. Army Medical Research Acquisition Activity through the Peer Reviewed Medical Research Program (grant number W81XWH-18-1-0717). The funders had no role in the design of the study; in the collection, analyses, or interpretation of data; in the writing of the manuscript, or in the decision to publish the results. 

## Conflict of Interest

The authors declare that the research was conducted in the absence of any commercial or financial relationships that could be construed as a potential conflict of interest.

## Publisher’s Note

All claims expressed in this article are solely those of the authors and do not necessarily represent those of their affiliated organizations, or those of the publisher, the editors and the reviewers. Any product that may be evaluated in this article, or claim that may be made by its manufacturer, is not guaranteed or endorsed by the publisher.

## Abbreviations

ACPAs, anti-citrullinated protein antibodies; CCP, cyclic citrullinated peptide; HLA, human leukocyte antigen; NETs, neutrophil extracellular traps; PAD, peptidylarginine deiminase; RA, rheumatoid arthritis; SE, shared epitope; SNP, single nucleotide polymorphism.
