# Supplementary material for: PADI4 Polymorphisms Confer Risk of Anti-CCP-Positive Rheumatoid Arthritis in Synergy With HLA-DRB1*04 and Smoking
Source: Front Immunol. 2021 Oct 18;12:707690. doi: 10.3389/fimmu.2021.707690 (PMC8558474; doi:10.3389/fimmu.2021.707690)
Supplement: Supplementary file 1 [file Image_1.pdf]

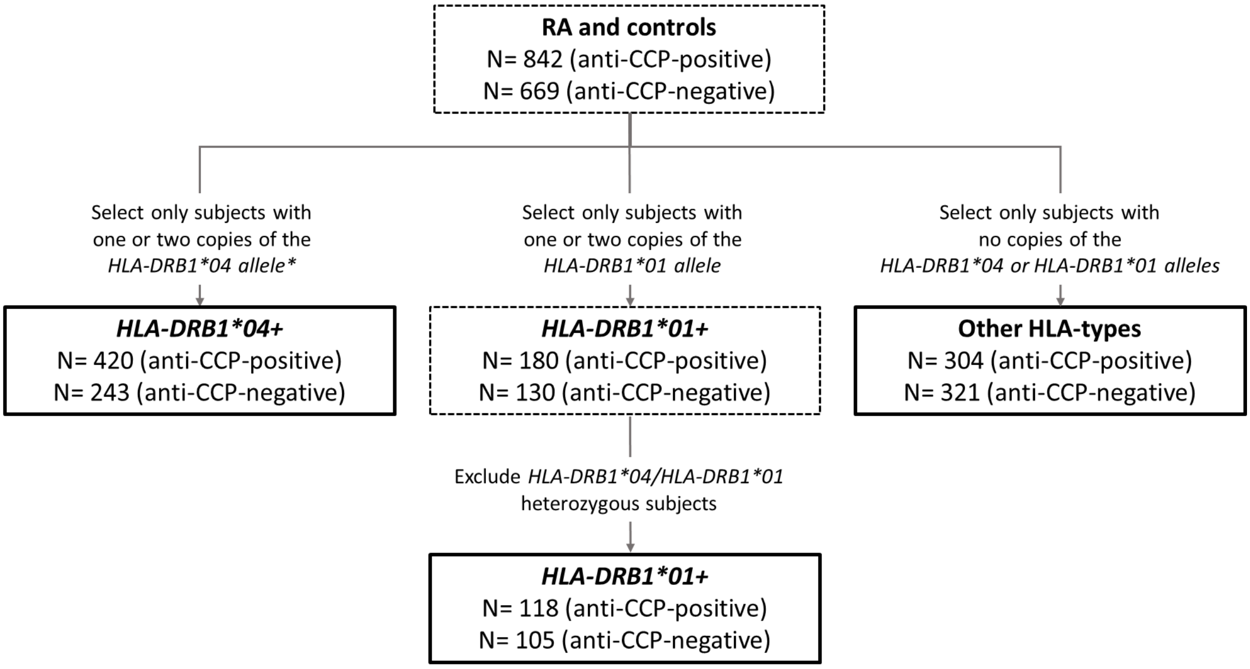

Supplementary figure 1: Procedure followed for the stratification of the Danish cohort based on HLA-type. Black full line boxes represented the three final groups for analyses presented in Table 3. *= grouping includes HLA-DRB1*04/HLA-DRB1*01 heterozygous subjects.
