# Supplementary material for: PADI4 Polymorphisms Confer Risk of Anti-CCP-Positive Rheumatoid Arthritis in Synergy With HLA-DRB1*04 and Smoking
Source: Front Immunol. 2021 Oct 18;12:707690. doi: 10.3389/fimmu.2021.707690 (PMC8558474; doi:10.3389/fimmu.2021.707690)
Supplement: Supplementary file 2 [file Table_1.docx]

| Supplementary table 1: Associations between selected polymorphisms in the *PADI4* gene and anti-CCP-positive or anti-CCP-negative RA in the Danish or North American cohorts. | | | | | | | | | | | | | | | | | | | | | |
| --- | --- | --- | --- | --- | --- | --- | --- | --- | --- | --- | --- | --- | --- | --- | --- | --- | --- | --- | --- | --- | --- |
|  |  |  | **Controls** | | |  | **Anti-CCP-positive RA** | | | | | | |  | **Anti-CCP-negative RA** | | | | | | |
| **Cohort** | **Minor allele** |  | **pp** | **pq** | **qq** |  | **pp** | **pq** | **qq** |  | **OR** | **95% CI** | **p-value** |  | **pp** | **pq** | **qq** |  | **OR** | **95% CI** | **p-value** |
| **Danish** | | | n=533 | | |  | n=309 | | |  |  |  |  |  | n=136 | | |  |  |  |  |
| rs74058715 | T |  | 465 | 67 | 1 |  | 278 | 31 | 0 |  | 0.77 | [0.49;1.22] | 0.27 |  | 128 | 8 | 0 |  | **0.41** | **[0.19;0.88]** | **0.02** |
| rs11203367 | T |  | 184 | 253 | 96 |  | 93 | 156 | 60 |  | 1.14 | [0.93;1.39] | 0.22 |  | 37 | 83 | 16 |  | 1.01 | [0.76;1.33] | 0.96 |
| rs1748033 | T |  | 250 | 237 | 46 |  | 136 | 136 | 37 |  | 1.16 | [0.93;1.44] | 0.18 |  | 55 | 72 | 9 |  | 1.11 | [0.82;1.49] | 0.50 |
| rs2240335 | A |  | 230 | 249 | 54 |  | 146 | 129 | 34 |  | 0.91 | [0.73;1.13] | 0.41 |  | 58 | 70 | 8 |  | 0.93 | **[0.69;1.26]** | 0.64 |
|  |  |  |  |  |  |  |  |  |  |  |  |  |  |  |  |  |  |  |  |  |  |
| **North American** cohort | |  | n=100 | | |  | n=104 | | |  |  |  |  |  | n=96 | | |  |  |  |  |
| rs74058715 | T |  | 88 | 12 | 0 |  | 91 | 12 | 1 |  | 1.16 | [0.51;2.60] | 0.73 |  | 92 | 4 | 0 |  | **0.30** | **[0.09;0.99]** | **0.05** |
| rs11203367 | T |  | 34 | 56 | 10 |  | 28 | 49 | 27 |  | **1.67** | **[1.10;2.56]** | **0.02** |  | 27 | 53 | 16 |  | 1.35 | [0.86;2.13] | 0.19 |
| rs1748033 | T |  | 47 | 44 | 9 |  | 42 | 50 | 12 |  | 1.23 | [0.80;1.90] | 0.34 |  | 37 | 47 | 12 |  | 1.30 | [0.84;2.02] | 0.24 |
| rs2240335 | A |  | 37 | 48 | 15 |  | 53 | 46 | 5 |  | **0.51** | **[0.32;0.81]** | **0.004** |  | 50 | 41 | 5 |  | **0.52** | **[0.33;0.82]** | **0.005** |
| CCP: Cyclic Citrullinated Peptide. RA= Rheumatoid Arthritis. p: major allele. q: minor allele. OR: Odds Ratio. 95%CI: 95% confidence interval. Values marked in bold indicate 95% CI excluding 1.00 and p<0.05. Logistic regression with adjustment for age. sex. ever smoking and carriage of PTPN22 R620W. | | | | | | | | | | | | | | | | | | | | | |
