# Supplementary material for: PADI4 Polymorphisms Confer Risk of Anti-CCP-Positive Rheumatoid Arthritis in Synergy With HLA-DRB1*04 and Smoking
Source: Front Immunol. 2021 Oct 18;12:707690. doi: 10.3389/fimmu.2021.707690 (PMC8558474; doi:10.3389/fimmu.2021.707690)
Supplement: Supplementary file 3 [file Table_2.docx]

| Supplementary Table 2: Logistic regression models for anti-CCP-positive and -negative RA, respectively, including *PADI4* SNPs and other potential risk factors as well as interactions that improved the models with statistical significance | | | | | | | | |
| --- | --- | --- | --- | --- | --- | --- | --- | --- |
|  |  | **Anti-CCP-positive RA** | | | | | | |
|  |  | **rs74058715 (C)** | | |  | **rs1748033 (T)** | | |
|  |  | **OR** | **95%CI** | **p-value** |  | **OR** | **95%CI** | **p-value** |
| Age |  | 0.99 | [0,98; 1.01] | 0.88 |  | 0.99 | [0.98;1.01] | 0.86 |
| Sex |  | **1.53** | **[1.08; 2.17]** | **0.02** |  | **1.56** | **[1.10;2.20]** | **0.01** |
| Ever smoker | | 1.18 | [0.74;1.89] | 0.48 |  | **1.85** | **[1.31;2.60]** | **<0.0001** |
| *HLA-DRB1*01* | | **2.37** | [1.60;3.52] | **<0.0001** |  | **2.36** | **[1.60;3.50]** | **<0.0001** |
| *HLA-DRB1*04* | | **4.41** | [2.61;7.46] | **<0.0001** |  | **4.40** | **[2.63;7.36]** | **<0.0001** |
| PTNPN22 R620W | | **3.04** | [1.78;5.17] | **<0.0001** |  | **2.87** | **[1.68;4.89]** | **<0.0001** |
| *PADI4* SNP | | 0.89 | [0.52;1.52] | 0.67 |  | 0.69 | [0.41;1.13] | 0.14 |
| *HLA-DRB1*04***PADI4* SNP | |  | - |  |  | **2.56** | **[1.34;4.88]** | **0.004** |
| *HLA-DRB1*04***PADI4* SNP*Ever smoker | | **2.29** | **[1.24;4.24]** | **0.008** |  |  | - |  |
|  |  |  |  |  |  |  |  |  |
|  |  | **Anti-CCP-negative RA** | | | | | | |
|  |  | **rs74058715 (C)** | | |  | **rs1748033 (T)** | | |
|  |  | **OR** | **95%CI** | **p-value** |  | **OR** | **95%CI** | **p-value** |
| Age |  | 1.01 | [0.99;1.03] | 0.36 |  | 1.01 | [0.99;1.03] | 0.40 |
| Sex |  | **2.28** | **[1.44;3.60]** | **<0.0001** |  | **2.23** | **[1.41;3.52]** | **0.001** |
| Ever smoker | | 1.24 | [0.83;1.85] | 0.29 |  | 1.05 | [0.68;1.62] | 0.81 |
| *HLA-DRB1*01* | | 1.07 | [0.66;1.74] | 0.77 |  | 1.11 | [0.68;1.80] | 0.67 |
| *HLA-DRB1*04* | | 1.05 | [0.70;1.57] | 0.83 |  | 0.76 | [0.45;1.28] | 0.30 |
| PTNPN22 R620W | | 1.23 | [0.78;1.94] | 0.37 |  | 1.24 | [0.78;1.95] | 0.36 |
| *PADI4* SNP | | **2.46** | **[1.14;5.27]** | **0.02** |  | 1.08 | [0.69;1.67] | 0.74 |
| *HLA-DRB1*04***PADI4* SNP | |  | - |  |  |  | - |  |
| *HLA-DRB1*04***PADI4* SNP*Ever smoker | |  | - |  |  | **2.19** | **[1.01;4.74]** | **0.05** |
| CCP: Cyclic Citrullinated Peptide, RA= Rheumatoid Arthritis, OR: Odds Ratio, 95%CI: 95% confidence interval. Values marked in bold indicate 95% CI excluding 1.00 and p<0.05. A full factorial set of interactions between the RA risk factors was presented to a logistic regression model. On grey background interaction between *PADI4* SNPs alleles and other RA risk factors, conditionally included only if they significantly improved the predictive performance of the model. | | | | | | | | |
|  |  |  |  |  |  |  |  |  |
|  |  |  |  |  |  |  |  |  |
|  |  |  |  |  |  |  |  |  |
