# Supplementary material for: PADI4 Polymorphisms Confer Risk of Anti-CCP-Positive Rheumatoid Arthritis in Synergy With HLA-DRB1*04 and Smoking
Source: Front Immunol. 2021 Oct 18;12:707690. doi: 10.3389/fimmu.2021.707690 (PMC8558474; doi:10.3389/fimmu.2021.707690)
Supplement: Supplementary file 4 [file Table_3.docx]

| Supplementary table 3: Risk of anti-CCP-positive RA, according to combinations of *HLA-DRB1*04*, *PADI4* SNPs, PTPN22 R620W and smoking status | | | | | | |
| --- | --- | --- | --- | --- | --- | --- |
|  |  |  |  |  | **Anti-CCP-positive RA** | |
|  |  |  |  |  |  |  |
| ***HLA-DRB1*04*** | **rs74058715(T)** | **PTPN22 R620W** | **Smoker** |  | **OR** | **p-value** |
|  |  |  |  |  |  |  |
| None | None | None | Never |  | 1.00 | 1.00 |
| Any | None | None | Never |  | **7.49** | **8.53E-06** |
| None | Any | None | Never |  | 1.90 | 0.46 |
| Any | Any | None | Never |  | **7.77** | **6.48E-04** |
| None | None | Any | Never |  | **4.81** | **7.47E-03** |
| Any | None | Any | Never |  | **11.53** | **1.49E-05** |
| None | Any | Any | Never |  | 2.14E-06 | 0.99 |
| Any | Any | Any | Never |  | 3.33 | 0.33 |
| None | None | None | Ever |  | 1.21 | 0.70 |
| Any | None | None | Ever |  | **17.10** | **2.52E-11** |
| None | Any | None | Ever |  | 2.81 | 0.17 |
| Any | Any | None | Ever |  | **5.25** | **9.08E-03** |
| None | None | Any | Ever |  | **6.93** | **1.78E-04** |
| Any | None | Any | Ever |  | **23.42** | **2.68E-11** |
| None | Any | Any | Ever |  | 2.33E-06 | 0.99 |
| Any | Any | Any | Ever |  | **9.35** | **4.41E-03** |
|  |  |  |  |  |  |  |
| ***HLA-DRB1*04*** | **rs11203367(T)** | **PTPN22 R620W** | **Smoker** |  | **OR** | **p-value** |
|  |  |  |  |  |  |  |
| None | None | None | Never |  | 1.00 | 1.00 |
| Any | None | None | Never |  | **6.33** | **0.03** |
| None | Any | None | Never |  | 1.67 | 0.54 |
| Any | Any | None | Never |  | **11.67** | **1.41E-03** |
| None | None | Any | Never |  | 1.99 | 0.60 |
| Any | None | Any | Never |  | **11.46** | **8.19E-03** |
| None | Any | Any | Never |  | **7.69** | **0.02** |
| Any | Any | Any | Never |  | **13.94** | **3.06E-03** |
| None | None | None | Ever |  | 2.54 | 0.27 |
| Any | None | None | Ever |  | **14.89** | **4.90E-04** |
| None | Any | None | Ever |  | 1.41 | 0.68 |
| Any | Any | None | Ever |  | **23.17** | **3.22E-05** |
| None | None | Any | Ever |  | **8.33** | **0.02** |
| Any | None | Any | Ever |  | **29.01** | **8.67E-05** |
| None | Any | Any | Ever |  | **7.49** | **0.02** |
| Any | Any | Any | Ever |  | **26.63** | **3.02E-05** |
|  |  |  |  |  |  |  |
| ***HLA-DRB1*04*** | **rs1748033(T)** | **PTPN22 R620W** | **Smoker** |  | **OR** | **p-value** |
|  |  |  |  |  |  |  |
| None | None | None | Never |  | 1.00 | 1.00 |
| Any | None | None | Never |  | **2.86** | **0.05** |
| None | Any | None | Never |  | 0.32 | 0.13 |
| Any | Any | None | Never |  | **5.52** | **1.15E-03** |
| None | None | Any | Never |  | 1.50 | 0.61 |
| Any | None | Any | Never |  | **4.25** | **0.03** |
| None | Any | Any | Never |  | 3.36 | 0.09 |
| Any | Any | Any | Never |  | **6.61** | **8.28E-03** |
| None | None | None | Ever |  | 1.00 | 1.00 |
| Any | None | None | Ever |  | **6.24** | **1.95E-04** |
| None | Any | None | Ever |  | 0.55 | 0.32 |
| Any | Any | None | Ever |  | **10.39** | **1.80E-06** |
| None | None | Any | Ever |  | **3.46** | **0.04** |
| Any | None | Any | Ever |  | **9.20** | **1.19E-04** |
| None | Any | Any | Ever |  | 2.79 | 0.14 |
| Any | Any | Any | Ever |  | **12.80** | **2.93E-06** |
| CCP: Cyclic Citrullinated Peptide, RA: Rheumatoid Arthritis, HLA: Human Leukocyte Antigen, OR: Odds Ratio. Logistic regression with adjustment for age and sex. | | | | | | |
